# Incorporating multiple biomarkers to assess mortality risk in non‐ST elevation myocardial infarction

**DOI:** 10.1002/clc.24300

**Published:** 2024-06-14

**Authors:** William Laband, Igor Vaz

**Affiliations:** ^1^ Department of Medicine St. Elizabeth's Medical Center Boston Massachusetts USA; ^2^ Division of Cardiology University of Pennsylvania Philadelphia Pennsylvania USA

To the Editor,

We read with keen interest the article “The CRP troponin test (CTT) stratifies mortality risk in patients with non‐ST elevation myocardial infarction” and commend the authors on their well‐conducted study.^1^

The utilization of biomarkers other than troponin for ACS risk stratification has gained traction in recent times, which is highlighted with the inclusion of CRP in the most recent version of the SMART2 risk prediction algorithm.^2^ Having said that, some studies have suggested that NT‐pro‐BNP, cTnT, and GDF‐15 have a stronger association with major adverse cardiac events than CRP.^3^ Ultimately, prediction scores incorporating multiple biomarkers may have the greatest clinical utility, and this study adds to the overall body of evidence.

Notably, in the article, the patients with the highest CTT levels were also less likely to receive angiography and experienced delays when it was performed.^1^ Exploring the reasons and implications of this would be beneficial. Additionally, given that the patients in the high CRP groups had a higher baseline creatinine and worse outcomes, this may have contributed to decision‐making and ultimately acted as a significant confounding factor.

## Data Availability

Data sharing is not applicable to this article as no new data were created or analyzed in this study.
